# Health Insurance Coverage and Hypertension Control in China: Results from the China Health and Nutrition Survey

**DOI:** 10.1371/journal.pone.0152091

**Published:** 2016-03-22

**Authors:** Yi Liao, Stuart Gilmour, Kenji Shibuya

**Affiliations:** Department of Global Health Policy, University of Tokyo, Tokyo, Japan; National Cardiovascular Center Hospital, JAPAN

## Abstract

**Background:**

China has rapidly expanded health insurance coverage over the past decade but its impact on hypertension control is not well known. We analyzed factors associated with hypertension and the impact of health insurance on the management of hypertension in China from 1991 to 2009.

**Methods and Findings:**

We used individual-level data from the China Health and Nutrition Survey (CHNS) for blood pressure, BMI, and other socio-economic variables. We employed multi-level logistic regression models to estimate the factors associated with prevalence and management of hypertension. We also estimated the effects of health insurance on management of hypertension using propensity score matching. We found that prevalence of hypertension increased from 23.8% (95% CI: 22.5–25.1%) in 1991 to 31.5% (28.5–34.7%) in 2009. The proportion of hypertensive patients aware of their condition increased from 31.7% (28.7–34.9%) to 51.1% (45.1–57.0%). The proportion of diagnosed hypertensive patients in treatment increased by 35.5% in the 19 years, while the proportion of those in treatment with controlled blood pressure remained low. Among diagnosed hypertensives, health insurance increased the probability of receiving treatment by 28.7% (95% CI: 10.6–46.7%) compared to propensity-matched individuals not covered by health insurance.

**Conclusions:**

Hypertension continues to be a major health threat in China and effective control has not improved over time despite large improvements in awareness and treatment access. This suggests problems in treatment quality, medication adherence and patient understanding of the condition. Improvements in hypertension management, quality of medical care for those at high risk, and better health insurance packages are needed.

## Introduction

In China, hypertension is the second most common disease risk factor [1]. Approximately 177 million people are estimated to be living with hypertension, with relatively low rates of awareness and control of this condition. [2,3] Previous studies have shown that public health services may be making little contribution in informing patients of their hypertensive status, [4] and more research is needed to understand how the response to this disease can be improved in the context of a rapidly changing health system and an aging population. Also, although some research on hypertension management in China has been conducted, the direct associations of health insurance on hypertension management still remain unknown.

China’s health insurance coverage has increased rapidly from 45% in 2006 to around 90% in 2009, [5] but the effectiveness of health insurance packages for hypertensive patients is not yet well understood at the population level, even though some contain special measures for severely hypertensive patients. [6] Given China’s rapid development over the past decade and the changes in health insurance coverage over the last five years, it is essential to evaluate the impact of health insurance on hypertension management and control.

In this article we had three main objectives:

- Estimating the prevalence of hypertension in China during the period of the CHNS;
- Estimating the proportion of those with prevalent hypertension who were aware of their disease, receiving treatment for it, or had controlled blood pressure;
- Estimating the effect of increased health insurance coverage on the management of hypertension.

## Method

### Data sources

We used datasets from the China Health and Nutrition Survey (CHNS), a cohort study established in 1989 with a total of eight survey waves in nine provinces until 2009. From 1989 to 2009, the CHNS collected data over a total of eight waves in nine provinces: Guangxi, Guizhou, Heilongjiang, Henan, Hubei, Hunan, Jiangsu, Liaoning, and Shandong. A stratified, multistage cluster sampling process was used to select samples in each of the provinces. Counties in each province were stratified according to income (low, middle, and high), and four counties in each province were selected using probability sampling. In each province, the capital city and a low-income city were selected if possible, although in two provinces large cities other than capitals had to be selected. Within these primary sampling units, villages, townships and urban/suburban neighborhoods were selected randomly. From 1989 to 1993 there were 190 primary sampling units: 32 urban neighborhoods, 30 suburban neighborhoods, 32 towns (county capital city), and 96 rural villages. After 2000, this increased to 216 units comprised of 36 urban neighborhoods, 36 suburban neighborhoods, 36 towns, and 108 villages. [7]

In this study, we used a repeated cross-sectional design, focusing on only new subjects above 35 years old in each wave for two reasons: First, physical exams in previous waves may have some effect on the awareness of high blood pressure in study subjects; secondly, the population under 35 has low prevalence of hypertension and hypertension in this age group may reflect very different risk factors. We excluded the first (1989) wave because the blood pressure testing method was different in this wave and information on some other socioeconomic variables was missing. Participants without fully recorded blood pressure testing results in other waves were also excluded in this study. For propensity score matching, we also took out participants who did not have socioeconomic variables that we used for matching.

### Statistical analysis

Multi-level logistic regression models were used in this analysis. We modeled a household-level random intercept to adjust for the genetic and socio-economic similarities within one family. The four outcomes in these models were:

1. *Prevalence*, defined as hypertension diagnosed at the time of entry into the study cohort or normal blood pressure readings in a subject currently taking anti-hypertensive medication. Hypertension was defined as average systolic blood pressure of the second and third of three tests≥140 mm Hg, or average diastolic blood pressure of the second and third test≥90 mm Hg;
2. *Awareness*, defined as a self-report of medically-diagnosed hypertension among subjects with hypertension;
3. *Treatment*, defined as people aware of their hypertension who were receiving anti-hypertensive medication treatment; and
4. *Control*, defined as normal blood pressure among people who were receiving treatment

Predictors tested in this model were sex, obesity, age and region of residence; study wave; education; income; presence of health insurance; smoking and alcohol use. Household per capita income in Yuan, inflated to 2009 values (1 US dollar = 6.83 Yuan), [8] was used as the income predictor in the model, divided into quintiles. Standardized prevalence of hypertension was also calculated to adjust for changes in the age distribution of new entrants over time. Since no information on probability weights is provided in the CHNS, no probability weights were used in this model. [7] We retained all possible predictors in our final regression model including those that were not statistically significant.

### Propensity score matching

We employed propensity score matching to estimate the average effects of health insurance on the management and control of hypertension. Propensity score matching is a process to adjust the data by matching the treated group to a control group that is defined as similar according to a propensity score developed based on background characteristics. [9] After propensity score matching, the comparability between the treatment variable (in this study, health insurance) and confounder variables is enhanced. By reducing the link between treatment variable and background variables through propensity score matching, regression results are less model-dependent and have reduced potential for bias. [9]

For the propensity score matching analysis, we used the k-Nearest neighbors matching method with five neighbors and matched individuals having health insurance with individuals who did not have health insurance using a propensity-score calculated based on study wave, age, sex, province, residency, income, and education level. One-to-one matching was used for sensitivity analysis, and found the similar results.

## Results

### Demographic characteristics of the sample

A total of 25,936 new subjects were involved in the CHNS until 2009. From these 1609 (6.2%) new participants in wave 1989 and 14,356 (55.4%) subjects aged under 35 or with no age information were removed. Finally, a total of 9971 (38.4%) subjects were used in our study with a mean age of 52.5 (range 35.0 to 96.5 years old). Table 1 shows the descriptive statistics of the study sample. The health insurance coverage rate in this sample was 37.4%, and 45.5% of the total sample came from the 1991 wave.

**Table 1:** Demographic and socio-economic properties of the sample.

	Frequency	Proportion (%)
Wave		
1991	4532	45.5
1993	414	4.2
1997	1705	17.1
2000	738	7.4
2004	1029	10.3
2006	475	4.8
2009	1078	10.8
Gender		
Male	4826	48.4
Female	5145	51.6
Province		
Liaoning	1113	11.2
Heilongjiang	914	9.2
Jiangsu	1160	11.6
Shandong	1293	13.0
Henan	1202	12.1
Hubei	874	8.8
Hunan	968	9.7
Guangxi	1297	13.0
Guizhou	1150	11.5
Residency		
Urban	3931	39.4
Rural	6040	60.6
Age		
35–44	3465	34.8
45–54	2572	25.8
55–64	2164	21.7
65–74	1221	12.3
≥75	549	5.5
BMI		
Normal	6488	65.1
Underweight	774	7.8
Overweight	1948	19.5
Obese	761	7.6
Smoking history		
None or past smoker	6470	66.1
Current smoker	3313	33.9
Alcohol use frequency		
None	6095	62.3
No more than 2 times a week	1948	19.9
More than 2 times a week	1738	17.8
Health insurance		
No	6221	62.6
Yes	3714	37.4
Education		
Not graduated from primary school	3729	38.6
Graduated from primary school	2138	22.1
Graduated from middle or high school	3042	31.5
Tertiary education	765	7.9

### Prevalence of hypertension

Table 2 shows prevalence of hypertension along with multi-level logistic regression analysis results. Prevalence of hypertension among the whole sample was 28.3% (95% CI 27.3–29.2%) and increased to 31.5% (28.5–34.7%) in 2009. Trends in prevalence of hypertension were unchanged after standardization using the WHO Standard Population as a reference distribution. Age was significantly positively associated with prevalence of hypertension, and more than half of elderly individuals (over 65 years old) had hypertension. Consistent with previous studies, women had lower prevalence of hypertension than men with odds ratio 0.67 (95% CI 0.57–0.79). Obesity increased the odds ratio of being hypertensive by a factor of 4.48 (3.41–5.89). Heavy drinking was also positively associated with prevalence of hypertension with an odds ratio of 1.34 (1.11–1.61). However, smoking and income had little effect. Compared to Liaoning province, Jiangsu, Hubei, and Guangxi provinces had lower prevalence of hypertension.

**Table 2:** Prevalence of hypertension by key risk factors.

	Frequency	Proportion (%)	Odds ratio (95% CI)	P-value
Age				
35–44	385	12.1	1	NA
45–54	566	23.7	2.41 (2.02–2.88)	<0.001
55–64	790	38.6	5.67 (4.67–6.88)	<0.001
65–74	605	52.4	12.10 (9.56–15.30)	<0.001
≥75	273	55.0	16.62 (12.23–22.58)	<0.001
Wave				
1991	1040	23.8	1	NA
1993	117	29.9	1.25 (0.92–1.71)	0.2
1997	501	33.5	1.38 (1.12–1.71)	0.003
2000	229	31.5	1.14 (0.87–1.50)	0.3
2004	311	32.5	1.20 (0.95–1.52)	0.1
2006	133	31.7	1.23 (0.90–1.66)	0.2
2009	288	31.5	1.36 (1.06–1.74)	0.02
Gender				
Male	1342	30.2	1	NA
Female	1277	26.5	0.66 (0.57–0.77)	<0.001
Province				
Liaoning	420	38.1	1	NA
Heilongjiang	284	36.9	0.99 (0.73–1.33)	0.9
Jiangsu	331	30.1	0.46 (0.36–0.60)	<0.001
Shandong	410	32.4	0.63 (0.50–0.81)	<0.001
Henan	286	28.5	0.58 (0.45–0.75)	<0.001
Hubei	234	28.1	0.65 (0.50–0.86)	0.002
Hunan	207	23.6	0.56 (0.43–0.74)	<0.001
Guangxi	230	19.3	0.35 (0.26–0.45)	<0.001
Guizhou	217	19.4	0.36 (0.27–0.47)	<0.001
Residency				
Urban	1225	33.3	1	NA
Rural	1394	24.9	0.90 (0.78–1.03)	0.1
BMI				
Normal	1456	23.3	1	NA
Underweight	160	21.3	0.58 (0.46–0.75)	<0.001
Overweight	788	41.6	2.39 (2.06–2.77)	<0.001
Obesity	225	56.1	4.25 (3.24–5.58)	<0.001
Smoke				
No or used	1754	29.0	1	NA
Current smoker	810	26.5	0.83 (0.72–0.97)	0.02
Alcohol use frequency				
No	1607	28.2	1	NA
No more than 2 times a week	428	24.1	0.93 (0.79–1.11)	0.4
More than 2 times a week	530	32.4	1.29 (1.07–1.54)	0.007
Have health insurance				
No	1431	24.6	1	NA
Yes	1181	34.5	1.15 (0.99–1.32)	0.06
Education				
No graduated from primary school	1070	30.5	1	NA
Graduated from primary school	517	25.9	0.90 (0.75–1.07)	0.2
Graduated from middle or school	700	25.2	1.04 (0.87–1.24)	0.7
Tertiary education	207	29.3	0.72 (0.55–0.94)	0.02
Quintiles of income				
1	486	22.5	1	NA
2	383	24.6	1.10 (0.90–1.36)	0.4
3	597	32.0	1.25 (1.03–1.52)	0.03
4	550	30.0	1.04 (0.85–1.28)	0.7
5	590	33.0	0.98 (0.78–1.25)	0.9

### Trends in factors associated with hypertension

**Fig 1:**
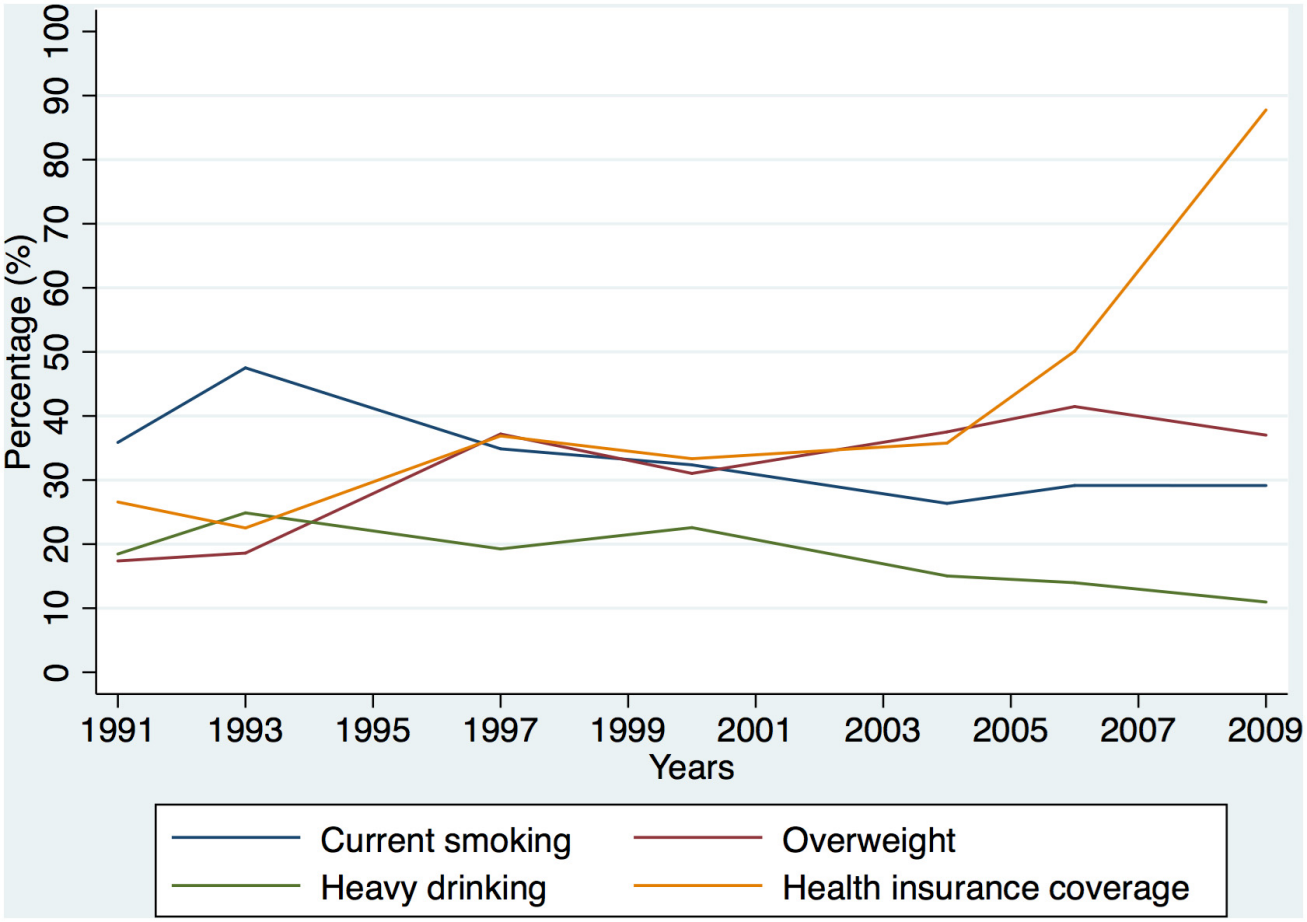
Trends in smoking, heavy drinking, obesity (including overweight and obesity) and health insurance coverage.

Fig 1 shows how trends in tobacco and alcohol use, BMI and health insurance coverage have changed over time in this survey. The smoking rate increased from 1991 to 1993, and gradually decreased after that. The obesity rate (including obesity and overweight) increased slightly from 1991 to 2009. Health insurance coverage increased over time and grew rapidly after 2006. In 2009, health insurance coverage reached around 90% in this sample.

### Management and control of hypertension

**Fig 2:**
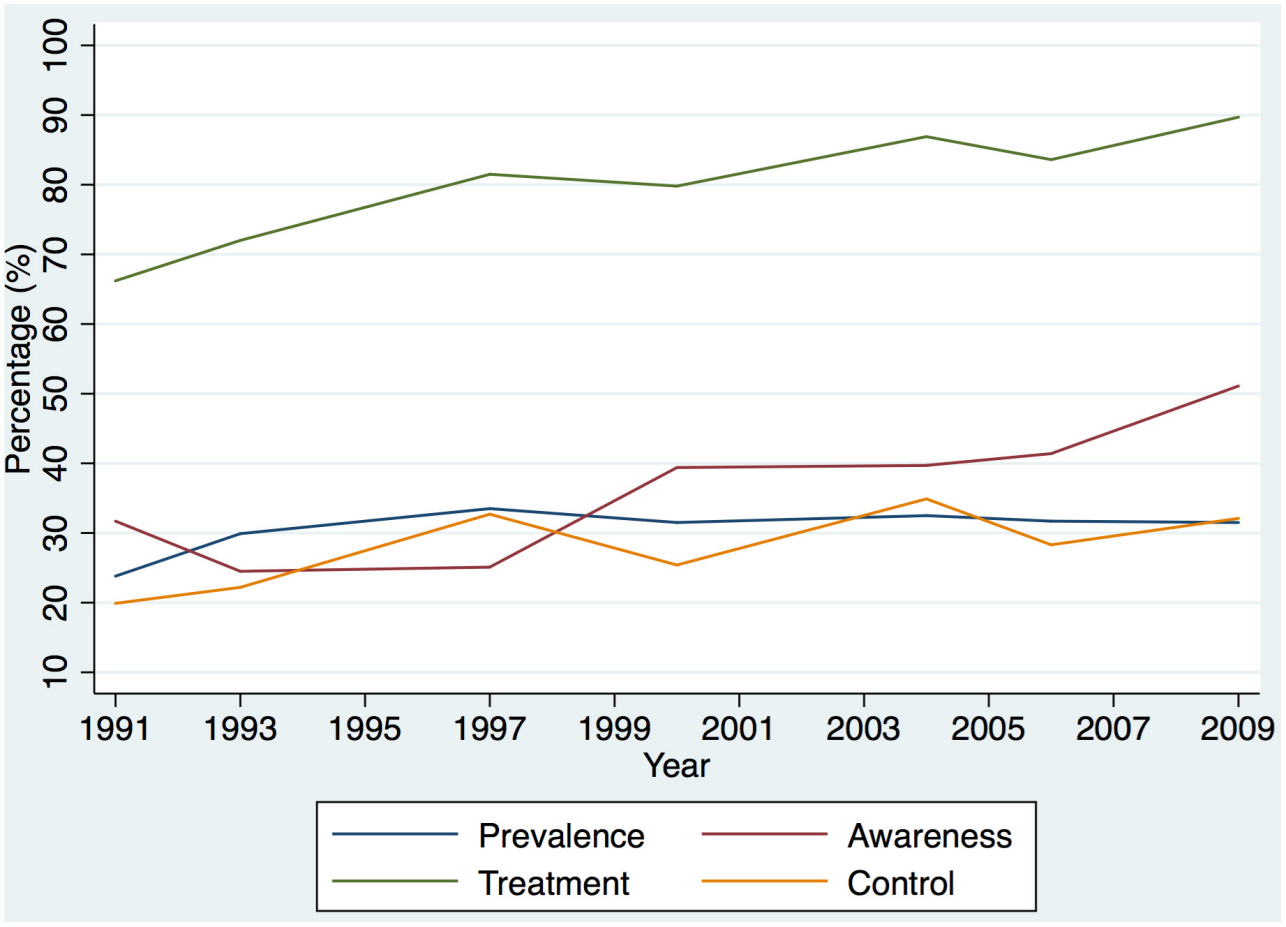
Trends in prevalence, awareness, treatment and control of hypertension.

Table 3 shows hypertension prevalence, awareness rates amongst hypertensives, treatment rates amongst diagnosed hypertensives, and control rates amongst medically-treated hypertensives in each wave. Fig 2 shows the trends in prevalence and outcomes of hypertension management over time. The proportion of people with hypertension who were aware of their condition gradually increased over time and reached 51.1% (95% CI 45.1–57.0%) in 2009 (Table 3). Table 4 shows the logistic regression results of management outcomes. Elderly, obese and well-educated subjects were more likely to be aware of their hypertension. Current smokers or people living in rural areas were less likely to know about their hypertension. Inequality in hypertension awareness rates was found among different provinces, with people from Jiangsu, Hubei, and Guangxi provinces more likely to be diagnosed (Table 4).

**Table 3:** Prevalence, awareness, treatment and control of hypertension.

Wave	Prevalence		Awareness		Treatment		Control	
	Frequency	Proportion (95% CI) (%)	Frequency	Proportion (95% CI) (%)	Frequency	Proportion (95% CI) (%)	Frequency	Proportion (95% CI) (%)
1991	1040	23.8 (22.5–25.1)	283	31.7 (28.7–34.9)	186	66.2 (60.3–71.7)	37	19.9 (14.4–26.4)
1993	117	29.9 (25.4–34.7)	25	24.5 (16.5–34.0)	18	72.0 (50.6–87.9)	4	22.2 (6.4–47.6)
1997	501	33.5 (31.1–36.0)	124	25.1 (21.3–29.1)	101	81.5 (73.5–87.9)	33	32.7 (23.7–42.7)
2000	229	31.5 (28.1–35.0)	89	39.4 (33.0–46.1)	71	79.8 (70.0–87.6)	18	25.4 (15.8–37.1)
2004	311	32.5 (29.6–35.6)	122	39.7 (34.2–45.5)	106	86.9 (79.6–92.3)	37	34.9 (25.9–44.8)
2006	133	31.7 (27.3–36.4)	55	41.4 (32.9–50.2)	46	83.6 (71.2–92.2)	13	28.3 (16.0–43.5)
2009	288	31.5 (28.5–34.7)	146	51.1 (45.1–57.0)	131	89.7 (83.6–94.1)	42	32.1 (24.2–40.8)
Total	2619	28.3 (27.3–29.2)	844	34.6(32.7–36.5)	659	78.3(75.3–81.0)	184	27.9 (24.5–31.5)

**Table 4:** Characteristics of management outcomes.

	Awareness		Treatment		Control	
	Odds ratio (95% CI)	P-value	Odds ratio (95% CI)	P-value	Odds ratio (95% CI)	P-value
Age						
35–44	1	NA	1	NA	1	NA
45–54	2.01 (1.37–2.95)	<0.001	1.85 (0.61–5.62)	0.3	0.44 (0.20–0.96)	0.04
55–64	3.53 (2.39–5.22)	<0.001	2.19 (0.77–6.26)	0.1	0.45 (0.22–0.95)	0.04
65–74	3.82 (2.51–5.80)	<0.001	1.31 (0.46–3.70)	0.6	0.49 (0.22–1.07)	0.07
≥75	2.95 (1.82–4.79)	<0.001	5.73 (1.05–31.30)	0.04	0.45 (0.18–1.13)	0.09
Wave						
1991	1	NA	1	NA	1	NA
1993	0.80 (0.45–1.46)	0.5	2.12 (0.45–9.99)	0.3	0.73 (0.18–3.00)	0.6
1997	0.57 (0.40–0.81)	0.002	2.88 (0.92–9.00)	0.07	1.43 (0.74–2.80)	0.3
2000	1.18 (0.76–1.84)	0.5	4.47 (1.22–16.32)	0.02	1.82 (0.78–4.25)	0.2
2004	1.11 (0.77–1.62)	0.6	4.75 (1.36–16.58)	0.02	2.03 (1.04–4.00)	0.04
2006	1.23 (0.77–1.98)	0.4	3.85 (0.91–16.23)	0.07	1.21 (0.52–2.84)	0.7
2009	1.90 (1.28–2.82)	0.001	3.78 (1.14–12.59)	0.03	1.71 (0.88–3.34)	0.1
Gender						
Male	1	NA	1	NA	1	NA
Female	1.35 (1.04–1.74)	0.02	1.18 (0.61–2.27)	0.6	0.79 (0.49–1.26)	0.3
Province						
Liaoning	1	NA	1	NA	1	NA
Heilongjiang	1.27 (0.80–2.03)	0.3	0.87 (0.26–2.77)	0.8	3.19 (1.30–7.82)	0.01
Jiangsu	1.87 (1.26–2.78)	0.002	4.99 (1.28–19.54)	0.02	2.65 (1.22–5.79)	0.01
Shandong	1.08 (0.74–1.58)	0.7	1.82 (0.66–5.02)	0.2	1.95 (0.84–4.56)	0.1
Henan	1.01 (0.66–1.55)	0.9	3.97 (1.01–15.55)	0.05	2.48 (1.05–5.89)	0.04
Hubei	1.89 (1.21–2.97)	0.005	2.67 (0.78–9.15)	0.1	3.49 (1.41–8.630)	0.007
Hunan	1.45 (0.91–2.32)	0.1	2.40 (0.69–8.34)	0.2	3.65 (1.41–9.44)	0.008
Guangxi	1.58 (1.01–2.46)	0.04	3.63 (0.91–14.40)	0.07	4.05 (1.69–9.72)	0.002
Guizhou	1.55 (0.96–2.51)	0.08	1.96 (0.54–7.17)	0.3	2.79 (1.09–7.13)	0.03
Residency						
Urban	1	NA	1	NA	1	NA
Rural	0.75 (0.60–0.95)	0.01	1.05 (0.58–1.91)	0.9	1.02 (0.68–1.56)	0.9
BMI						
Normal	1	NA	1	NA	1	NA
Underweight	0.50 (0.29–0.84)	0.01	4.22 (0.66–26.86)	0.1	0.83 (0.28–2.48)	0.7
Overweight	1.39 (1.10–1.76)	0.005	1.57 (0.84–2.95)	0.2	0.62 (0.40–0.96)	0.03
Obesity	3.04 (2.07–4.45)	<0.001	5.07 (1.29–19.84)	0.02	1.51 (0.89–2.58)	0.1
Smoke						
No or used	1	NA	1	NA	1	NA
Current smoker	0.71 (0.54–0.93)	0.01	1.00 (0.50–2.01)	1	0.86 (0.50–1.46)	0.6
Alcohol use frequency						
No	1	NA	1	NA	1	NA
No more than 2 times a week	0.92 (0.68–1.26)	0.6	1.19 (0.52–2.75)	0.7	0.81 (0.45–1.44)	0.5
More than 2 times a week	0.74 (0.54–1.02)	0.07	0.59 (0.24–1.44)	0.2	0.85 (0.43–1.67)	0.6
Have health insurance						
No	1	NA	1	NA	1	NA
Yes	1.02 (0.79–1.31)	0.9	2.81 (1.22–6.46)	0.02	0.65 (0.40–1.07)	0.09
Education						
No graduated from primary school	1	NA	1	NA	1	NA
Graduated from primary school	1.58 (1.16–2.15)	0.003	1.39 (0.61–3.14)	0.4	0.81 (0.45–1.45)	0.5
Graduated from middle or school	1.52 (1.11–2.09)	0.009	0.80 (0.34–1.88)	0.6	1.51 (0.85–2.69)	0.2
Tertiary education	1.85 (1.19–2.86)	0.006	2.88 (0.74–11.31)	0.1	1.59 (0.78–3.26)	0.2
Quintiles of income						
1	1	NA	1	NA	1	NA
2	0.89 (0.60–1.31)	0.6	0.88 (0.33–2.32)	0.8	1.39 (0.59–3.29)	0.5
3	0.97 (0.69–1.37)	0.9	1.32 (0.54–3.25)	0.5	1.16 (0.55–2.46)	0.7
4	1.12 (0.79–1.60)	0.5	1.09 (0.45–2.64)	0.8	1.82 (0.90–3.65)	0.1
5	0.98 (0.66–1.45)	0.9	1.52 (0.52–4.49)	0.4	1.56 (0.73–3.32)	0.3

Among individuals who were diagnosed as hypertensive, treatment rates have increased rapidly from 66.2% (60.3–71.7%) to 89.7% (83.6–94.1%) over the 19 years (Table 3). Although treatment rates increased rapidly over time, only 32.1% (95% CI 24.2–40.8%) of treated people had their hypertension under control in the latest survey in 2009 (Table 3). Table 4 shows that only age and district were significantly associated with control, with older people or residents of Liaoning and Shandong provinces less likely to have their hypertension under control. There was no significant time trend in control rates amongst treated patients, and increased health insurance coverage also had little association with control.

### Propensity score matching

The average treatment effects of health insurance on awareness in total were 0.05 (95% CI -0.08 to 0.19, p-value 0.2), 0.06 (-0.08 to 0.20, p-value 0.2) in urban areas and 0.01 (-0.21 to 0.23, p-value 0.5) in rural areas after propensity score matching. The average treatment effect of health insurance on treatment was 0.29 (0.11 to 0.47, p-value 0.001) in total, 0.27 (0.05 to 0.48, p-value 0.008) in urban areas and 0.27 (-0.11 to 0.65, p-value 0.08) in rural areas. The average treatment effect of health insurance on control was -0.12 (-0.32 to 0.09, p-value 0.9) in total, -0.07 (-0.30 to 0.16, p-value 0.7) in urban areas and -0.05 (-0.41 to 0.30, p-value 0.6) in rural areas. Health insurance showed a positive effect only on treatment, people with health insurance had a 29% higher chance to receive hypertension treatment compared to those who do not have health insurance. The effect on awareness and control was not significant, which was consistent with the logistic regression results.

Among diagnosed hypertensives, health insurance increased the probability that they would receive treatment by 28.7% (95% CI: 10.6–46.7%, p-value 0.001) compared to propensity-matched individuals who were not covered by health insurance.

## Discussion

This is the first population-based study to assess trends in the management and control of hypertension using a household survey covering 19 years of rapid change in China, which used a similar sampling and data collection method for seven consecutive waves. Trends in prevalence and management of hypertension were comparable in each wave, along with their risk factors, so the results are comparable over time and enable comparison of the periods before and after coverage of insurance increased. This is also the first study to estimate the treatment effects of health insurance on prevalence and management of hypertension in China using propensity score matching.

Prevalence of hypertension reached about 30% after 1997, which is slightly higher than found in a cross-sectional study by Gao and colleagues in 2013 based on data collected from the 2007–2008 China National Diabetes and Metabolic Disorders Study. [10] This may be because of different definitions of hypertension in these two studies. Our method of defining hypertension was slightly less restrictive than that of Gao and colleagues, and may give increased prevalence estimates relative to their findings.

According to the latest Chinese guidelines for the management of hypertension, the prevalence of hypertension has significantly increased over time. [3] However, in this study, the time trend remains stable, which may be explained in two ways. First, the data used in the 2010 Chinese guidelines came from four surveys, with different study populations, methods and definitions. Second, we only used new participants in the CHNS, who have a similar age structure in each wave. Since age is the most important determinant of prevalence, similar age structures may result in a non-significant time trend while in other surveys, the age structure is changing in the study sample along with the aging process nationwide.

Consistent with previous studies, elderly men were at higher risk of hypertension, and obesity and heavy drinking had a strong positive effect on prevalence of hypertension. [4] People with middle incomes were more likely to have hypertension, which may be attributed to their lifestyles. The CHNS study does not include comprehensive information on salt intake, but according to previous studies, salt is widely used in home cooking in China. [11] Daily salt intake is generally higher in northern areas, and more than 80% of residents take salt in excess of dietary recommendations. [12] Unobserved differences in salt intake may explain regional or wealth-based differences in hypertension in this study. Smoking rates slightly decreased over time in this study, consistent with previous national surveys. [13] Also, consistent with previous studies, the prevalence of overweight and obesity in our study increased rapidly with rapid development in the economy and living standards in China. [14]

The awareness rate increased over time, and in the 2009 wave half of people who had hypertension were diagnosed. Although elderly and overweight people had greater awareness of their hypertensive status, the rate of awareness among these groups is still low given their much higher risk of hypertension. People living in rural areas and with lower education levels are less likely to be aware of their hypertension. This may be because these people lack the resources to obtain hypertension knowledge. Health education programs for elderly and rural populations may be effective ways to promote awareness of hypertension. [15] Differences in hypertension awareness between rural and urban areas may also relate to different types of health insurance targeting different populations. For instance, the New Rural Cooperative Medical Scheme (NCMS) covers people in rural areas while the Urban Resident/Employee Basic Medical Insurance covers people in urban areas. Our results indicate that there might be some inequity between urban and rural health insurance packages in early diagnoses of hypertension. Inequities in service utilization and health outcomes between urban and rural areas have been reported, especially in 1990s. [16]

Treatment rates amongst those diagnosed with hypertension increased to around 90% by 2009. However, among people receiving medication, the proportion of people who have actually achieved control of their hypertension was only 30%. This situation of low control of hypertension is consistent with previous studies both in China and other countries, which found limited hypertension-related knowledge, ignorance of non-pharmacological treatments, unsatisfactory medication adherence, and inappropriate medical treatment all limited the effectiveness of treatments. [3,17,18]

Although elderly people were at higher risk of hypertension, they were less likely to have controlled hypertension. This may be because systolic hypertension due to age-related factors such as arteriosclerosis was common among elderly people. [19] Previous studies have shown that it is more difficult to control systolic blood pressure than diastolic blood pressure, and combinations of two or more antihypertension drugs are required for effective treatment. [20] Also, inequalities in hypertension prevalence and management were found across provinces, which may be due to variation in lifestyles, dietary habits, health treatment costs, insurance reimbursement levels, and health promotion programs between different areas. However, there was not enough information about health policies and programs implemented at the sub-national levels, and more information and surveys are needed to explain this variation. Since prevention and management of hypertension are national health promotion goals, the inequalities across provinces indicate that stronger national-level monitoring and guidelines may be necessary to achieve national goals for hypertension control programs and policies.

Although increased health insurance coverage was significantly associated with increased levels of treatment, we found little effect of health insurance on control of hypertension. Some longitudinal studies in the US have shown a positive effect of health insurance on awareness and control of hypertension as well as drug adherence. [21] At the individual level, better health knowledge has been shown to be effective in promoting medication adherence and blood pressure control. [22] These findings indicate that there may be problems with the quality of treatment being received, access to drugs or adherence to management guidelines, together with personal health-seeking behavioral factors in China.

Hypertension, like other chronic diseases, needs long periods of outpatient treatment. However, some studies show that current Chinese medical benefit packages are still limited and mainly focus on inpatient costs. [23] A study in some provinces in China found that outpatient care for people with chronic diseases had very low reimbursement levels which indicates that people living with hypertension may receive inadequate financial support. [24] This could affect medication adherence since previous studies have shown a positive association between lower cost of medication and better medication adherence. [25]

Although primary health centers have been proven effective in early diagnosis and management of hypertension, there are no systematic guidelines and rules for hypertensive case management at the community level in China, which may lead to low medication adherence among hypertensive patients. Some community-level studies conducted in China have shown that adherence with drug regimens is lower than 50%. [26] Regular health checks and blood pressure tests are not implemented or included in the health insurance package nationally, although some studies have shown their effectiveness in early diagnosis and management of hypertension. [27]

The main limitation of this study is that no probability sampling information is available in the CHNS. [7] This will affect the accuracy of trend analysis, but has little effect on logistic regression analysis since we used sub-samples instead of the whole sample, and merged samples in different waves, making it impossible to apply sampling weights even where they are available. Provinces involved in this study were mostly located in the northeast and middle part of China, so there were no samples from the richest and poorest provinces. However, the nine provinces chosen in this study range from the wealthiest to the poorest five, and may be relatively representative of the national income distribution. [28] Another limitation is lack of information about the method used to measure blood pressure, which may vary by wave and district. We did not include the types of health insurance in our models because the proportion of missing data on this information is too high. Also the variables we used in this study are not comprehensive, and other unobserved characteristics such as physical activity could also effect the management of hypertension. Last but not least, there is no detailed information on the methods used to recruit new participants in the CHNS, so the new households added in the sample units might not be randomly selected, although these new units should be randomly selected.

This study suggests that China’s health insurance system has been effective in increasing hypertension treatment but that more needs to be done to improve effective coverage of hypertension control. Primary health care center involvement in health education, regular community-based screening, especially for high risk populations such as elderly men, and promotion of medication adherence should be considered where these interventions can be shown to be cost-effective. Also, lifestyle interventions such as reducing daily salt intake that have been proven effective in reducing blood pressure in Japan, [29] should also be considered in China. By improving public health and preventive measures in the current health insurance package, the Chinese government can act early and effectively to prevent the most serious consequences of this challenging and growing non-communicable disease epidemic.

## Supporting Information

S1 Appendix Detailed information and explanation on propensity score matching. (PDF)

S1 Fig Trends in age-standardized prevalence of hypertension. (PDF)

S1 Tables Age, gender and residency distribution over time. (PDF)
