# Supplementary material for: Health Insurance Coverage and Hypertension Control in China: Results from the China Health and Nutrition Survey
Source: PLoS One. 2016 Mar 22;11(3):e0152091. doi: 10.1371/journal.pone.0152091 (PMC4803201; doi:10.1371/journal.pone.0152091)
Supplement: S1 Appendix — (PDF) [file pone.0152091.s001.pdf]

S1 Appendix: Detailed information and explanation on propensity score matching.

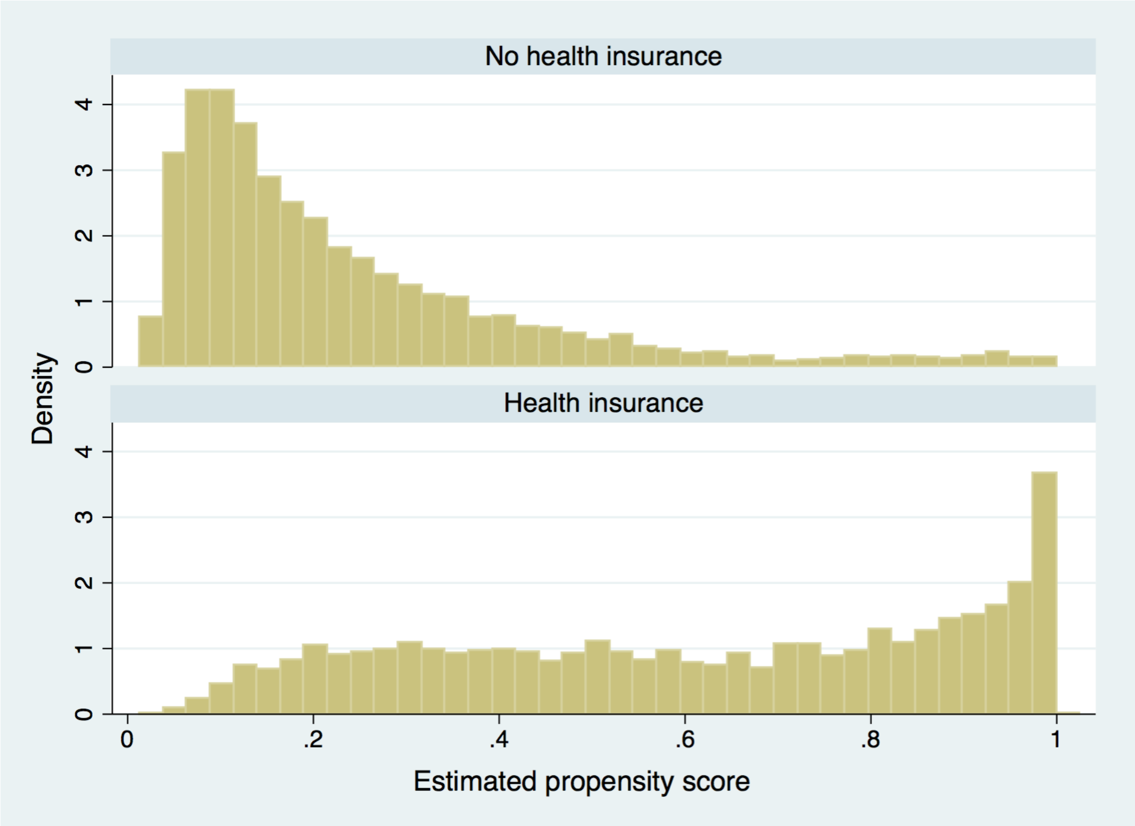

Fig 1. Distribution of propensity scores by health insurance situation.

		Off common support	On common support	Total
Awareness	Untreated	1,030	115	1,145
	Treated	827	261	1,088
Total		1,857	376	2,233
Treatment	Untreated	280	56	336
	Treated	337	101	438
Total		617	157	774
Control	Untreated	186	49	235
	Treated	306	71	377
Total		492	120	612

Table A. Degree of propensity score matching by treatment condition for each analysis.

The distribution is more skewed to the left in the case of participants without health insurance, while the distribution is generally uniform and more skewed to the right among individuals with health insurance. However, because of the wide range of distribution in participants with health insurance, the region of overlap is acceptable. In addition, from the figure we can see that individuals with and without health insurance are different. This may be because of the differences in health insurance coverage over time. By dropping individuals with bad balancing, the results in this study are exclusively for the comparison between participants with and without health insurance who had similar overall characteristics. Table 3 above shows the number of individuals on/off common support. One approach to ensure comparability is to focus on individuals lying in the common support of propensity score. However, this would significantly reduce the sample size and entail excluding treated individuals with propensity scores that are larger than the maximum propensity score observed in the untreated group. Given this, we did not impose the common support restriction in this study.
