# Supplementary material for: Health Insurance Coverage and Hypertension Control in China: Results from the China Health and Nutrition Survey
Source: PLoS One. 2016 Mar 22;11(3):e0152091. doi: 10.1371/journal.pone.0152091 (PMC4803201; doi:10.1371/journal.pone.0152091)
Supplement: S1 Fig — (PDF) [file pone.0152091.s002.pdf]

S1 Figure A: Trends in age-standardized prevalence of hypertension.

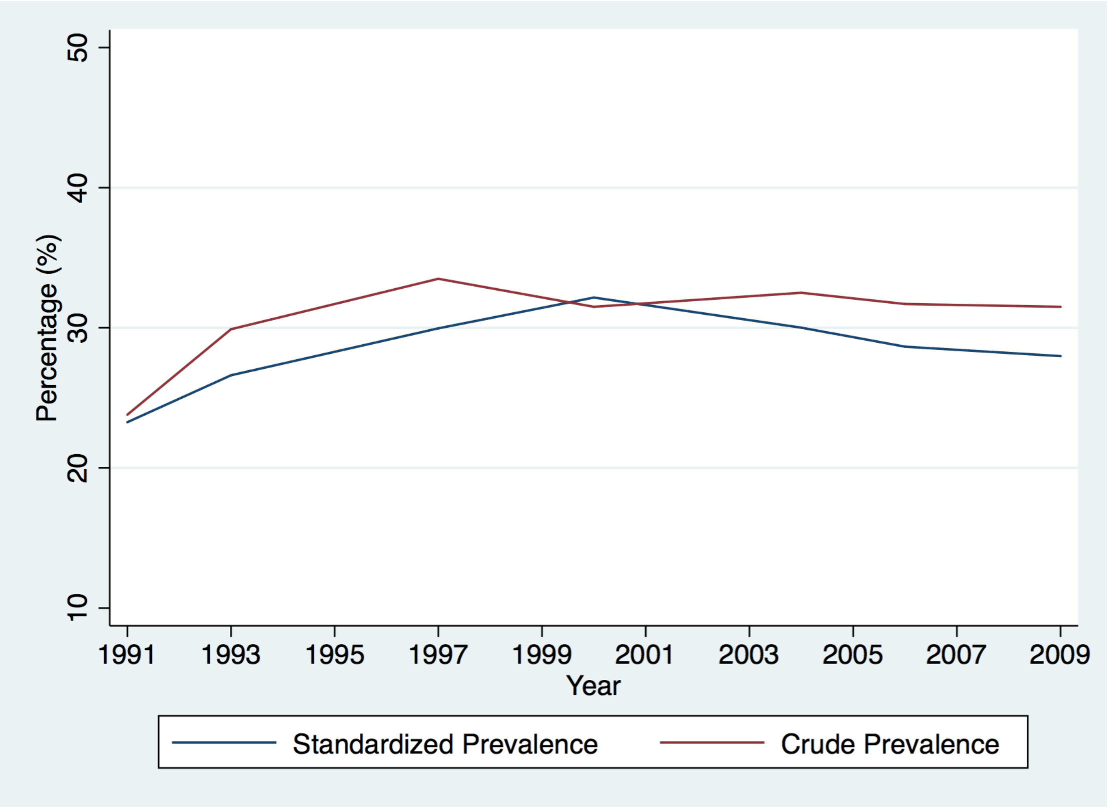

Figure A: Trends in age-standardized prevalence of hypertension.
