# Supplementary material for: Health Insurance Coverage and Hypertension Control in China: Results from the China Health and Nutrition Survey
Source: PLoS One. 2016 Mar 22;11(3):e0152091. doi: 10.1371/journal.pone.0152091 (PMC4803201; doi:10.1371/journal.pone.0152091)
Supplement: S1 Tables — (PDF) [file pone.0152091.s003.pdf]

S1 Table A and B: Age, gender and residency distribution over time.

Wave	35-44 years		45-54 years		55-64 years		65-74 years		≥75 years	
	Frequency	Proportion	Frequency	Proportion	Frequency	Proportion	Frequency	Proportion	Frequency	Proportion
1991	1535	33.9	1146	25.3	1078	23.8	540	11.9	233	5.1
1993	136	32.9	88	21.3	94	22.7	65	15.7	31	7.5
1997	606	35.5	459	26.9	356	20.9	197	11.6	87	5.1
2000	247	33.5	214	29.0	157	21.3	86	11.7	34	4.6
2004	353	34.3	270	26.2	188	18.3	145	14.1	73	7.1
2006	191	40.2	94	19.8	86	18.1	75	15.8	29	6.1
2009	397	36.8	301	27.9	205	19.0	113	10.5	62	5.8
Total	3465	34.8	2572	25.8	2164	21.7	1221	12.3	549	5.5

Table A. Age distribution over time.

	1991	1993	1997	2000	2004	2006	2009
	Frequency (Proportion %)	Frequency (%)	Frequency (%)	Frequency (%)	Frequency (%)	Frequency (%)	Frequency (%)
Gender							
Male	2130 (47.0)	242 (58.5)	871 (51.1)	355 (48.1)	483 (46.9)	231 (48.6)	514 (47.7)
Female	2402 (53.0)	172 (41.6)	834 (48.9)	383 (51.9)	546 (53.1)	244 (51.4)	564 (52.3)
Residency							
Urban	1319 (29.1)	115 (27.8)	886 (52.0)	359 (48.6)	501 (48.7)	212 (44.6)	539 (50.0)
Rural	3213 (70.9)	299 (72.2)	819 (48.0)	379 (51.4)	528 (51.3)	263 (55.4)	539 (50.0)

Table B. Gender and residency distribution by wave
